# A lens on salutogenic concepts as resources for oral health among adults: a scoping review

**DOI:** 10.1186/s12903-026-10341-2

**Published:** 2026-11-12

**Authors:** M. Nordström, E. Shmarina, U. Lindmark

**Affiliations:** 1 Faculty of Odontology, Malmö University, Malmö, Sweden; 2 Kalmar County Council, Public Dental Service, Oskarshamn, Sweden; 3 Department of Health Sciences, Karlstad University, Karlstad, Sweden

**Keywords:** Salutogenesis, Oral health, Health promotion

## Abstract

**Background:**

To effectively address the global burden of oral health disease, a broader and more sustainable approach focusing of health factors is suggested. The salutogenic approach, which emphasizes psychosocial health resources, offers a promising framework for health promotion. However, there is currently a lack of synthesized evidence on how salutogenic concepts are applied in adult oral health. This scoping review therefore aims to identify and map the existing scientific research on the use of salutogenic concepts as oral health resources among adults.

**Methods:**

This scoping view followed established guidelines. A systematic search was conducted across four databases (PubMed, Web of Science, PsycInfo, and CINAHL) using a search string strategy based on three key elements: Population (adults aged 18–59), Concept (salutogenic concepts), and Context (oral health). The outcomes of interest included dental caries, periodontitis, and self-reported oral health. A total of 2,503 articles were identified and screened for eligibility, followed by data extraction from the relevant studies.

**Results:**

A total of 28 studies were included in the review, most of which were cross-sectional and published between 1996 and 2025. The studies were conducted across multiple regions worldwide. Based on the salutogenic umbrella framework, 12 key concepts were identified: self-efficacy, sense of coherence, social capital, coping, locus of control, social support, optimism, emotional intelligence, motivation, positive emotionality, resilience, and well-being. These studies varied in how these concepts were defined, the instruments used, the populations studied, and the settings in which the research was conducted.

**Conclusions:**

There is a growing research interest in applying salutogenic approaches to adult oral health, although concentrated on a few salutogenic constructs and inconsistent in measurement. The findings highlight the relevance of salutogenic concepts for oral health promotion research. Future research using well-defined concepts and robust methodological designs is needed to strengthen the evidence base.

**Supplementary Information:**

The online version contains supplementary material available at https://doi.org/10.1186/s12903-026-10341-2.

## Introduction

While the interconnection between oral and general health is well established in dental public health, research remains predominantly focused on biological risk factors and disease outcomes. Incorporating a health-promoting perspective acknowledges the influence of social determinants, including psychosocial and environmental factors. This offers a more comprehensive framework for understanding and improving oral health [1, 2].

The salutogenic approach emphasizes health-promoting resources and explores why some people are able to maintain or improve their healthy despite facing stress and adversity – focusing on the origins of health [3, 4]. This approach aligns closely with health promotion and provides a theoretical foundation for practical interventions, as outlined in the WHO Ottawa Charter [4–6], which advocates for making healthy choices easy the choices [7].

There is a growing call for salutogenic research to complement the traditional pathogenic approach, which primarily focuses on risk factors [8]. The salutogenic perspective encourages viewing professional practice through a “health-positive paradigm or philosophical lens” [9]. It has been proposed as a valuable framework for promoting oral health at both individual and societal levels [10–13].

The salutogenic theory is often described as an umbrella framework that encompasses several related salutogenic concepts representing psychosocial factors as resources, competencies, or assets that are essential to explain positive health development and wellbeing [14]. The first and most central of these is Sense of Coherence (SOC), introduced in the literature as a life orientation that enables individuals to perceive life as meaningful, comprehensible, and manageable [4]. Since its initial application to oral health in 2001 [15, SOC has been studied across diverse populations and contexts. Systematic reviews have explored its associations with oral health outcomes and behaviors in children and adolescents [16–18], as well as its relevance to oral health-related quality of life [19] and dental fear or anxiety [20].

However, salutogenesis extends beyond SOC, and only a limited number of studies have explored a broader range of concepts described under the salutogenic umbrella framework. Concepts such as empowerment, resilience, locus of control, coping, social capital, social support, and self-efficacy are also considered as part of the salutogenic theory [14]. While SOC remains the central construct in Antonovsky’s original salutogenic theory, these related salutogenic concepts have been described as health-promoting resources that align with a broader salutogenic perspective. While these concepts share certain similarities, they also differ in important ways. Together, they offer complementary perspectives that are valuable for health promotion efforts focusing on strengthening the health resources of individuals, groups, and communities [3, 14]. Previous reviews of salutogenic concepts in oral health research have predominantly focused on their role as protective psychosocial factors, particularly in relation to children and adolescents [21, 22]. In contrast, few reviews have studied these concepts in adult populations, and those that do tend to focus mainly on SOC [23–26]. Currently, there is a lack of comprehensive compilations that explore the full range of salutogenic concepts in adult populations.

Considering the increased interest in oral health promotion and the emergence of new action plans aimed to improve oral health for all [27], this scoping review seeks to summarize the scientific literature on salutogenic concepts as resources for oral health, in relation to dental caries, periodontitis, and subjective oral health. This overview will give an understanding why some individuals maintain good oral health while others do not, thereby offering valuable insights for developing effective oral health promotion strategies. This scoping review therefore aims to identify and map the existing scientific research on the use of salutogenic concepts as oral health resources among adults.

### Specific research question

How are salutogenic concepts utilized in oral health research?

## Materials and methods

### Study type

This scoping review follows the guidelines as provided by the Joanna Briggs Institute [28]. In collaboration with a university librarian, a strategy search was developed based on the Population, Concept, and Context framework (PCC). The complete search strings are available in the Supplementary Material.

- P (Population): Adults aged 18-59 years
- C (Concept): salutogenic concepts as health resources, based on the salutogenic umbrella framework by Lindström, Eriksson (2010) [14]
- C (Context): Oral health, including both objective (dental caries and periodontitis) and subjective oral health-related outcomes

This study is reported in accordance with the PRISMA-ScR (Preferred Reporting Items for Systematic Reviews and Meta-Analyses extension for Scoping Reviews) Checklist [29]

### Eligibility criteria

- Studies utilizing salutogenic concepts as defined by the salutogenic umbrella framework [14].
- Adult population aged 18–59 years.
- Original quantitative studies, including cohort, case–control, cross-sectional, and intervention studies.
- Published in peer-reviewed academic journals.
- Reporting explicit oral health-related outcomes, either objective (e.g., dental caries, periodontitis) or subjective (self-perceived/self-reported oral health).
- Conducted in any setting, such as dental clinics, households, workplaces, or universities.
- Studies published up to February 2025.

### Exclusion criteria

Grey literature was excluded in this review as the search was limited only to peer-reviewed articles. To maintain a methodologically coherent age span, studies involving participants outside the age range 18–59 years were excluded, unless the results were age stratified. This criterion minimized confounding from developmental and age-related physiological differences. Additionally, studies using Quality of Life (QoL) as an oral health-related outcome, but not linking it to any salutogenic concepts were not considered and thus excluded. No restrictions were applied regarding the year of publication or language.

### Search strategy and study selection

Database searches were conducted on October 3, 2023, using PubMed, Web of Science, PsycInfo. In February 2025, PubMed was searched, as this database was deemed to provide the most extensive coverage of the research area and had yielded the majority of studies in the original search. Only studies published up to February 2025 were eligible for inclusion in the review. Reference lists were hand-searched independently by the two authors, MN and ES.

PubMed and CINAHL were chosen as core databases for health science, while PsycInfo was included for its coverage of behavioral and social literature. Scopus and Web of Science were also used due to their extensive coverage of multidisciplinary literature. Covidence was employes as a data management and extraction tool, providing a consistent workflow that minimizes the risk of human error and bias [30].

An assessment of methodological quality was not conducted, as it is not generally recommended in scoping reviews. The primary aim is to map descriptive characteristics rather than to provide clinically meaningful conclusions. To ensure consistency, all authors first calibrated by jointly screening 15 titles and abstracts. The remaining titles and abstracts were then screened independently by MN and ES. The same procedure was applied for full text review: following an initial calibration, ES and MN screened the texts independently, and any disagreements were resolved through discussion and consensus with UL.

A priori scoping review protocol was created to guide the data extraction, however, the protocol was not formally published. It was piloted by all authors and then modified to finally include sample, age, total number, study design, setting, aim, outcome measures, salutogenic concepts, instruments and concluding results. Data extraction was done independently by MN and ES, followed by comparison and, when needed, consensus discussions with UL. If any information could not be retrieved from an article during screening or extraction, the relevant authors were contacted.

### Analysis and presentation of results

The categorization process involved iterative discussion among the review team to ensure consistency and theoretical coherence. The salutogenic concepts were categorized according to the salutogenic umbrella framework [14] and listed in descending order based on the number of studies. Health outcomes were grouped into two categories: objective (periodontal disease, dental caries) and subjective oral health (self-reported oral health, also referred to as self-rated and self-perceived oral health).

## Results

### Study selection

**Fig. 1:**
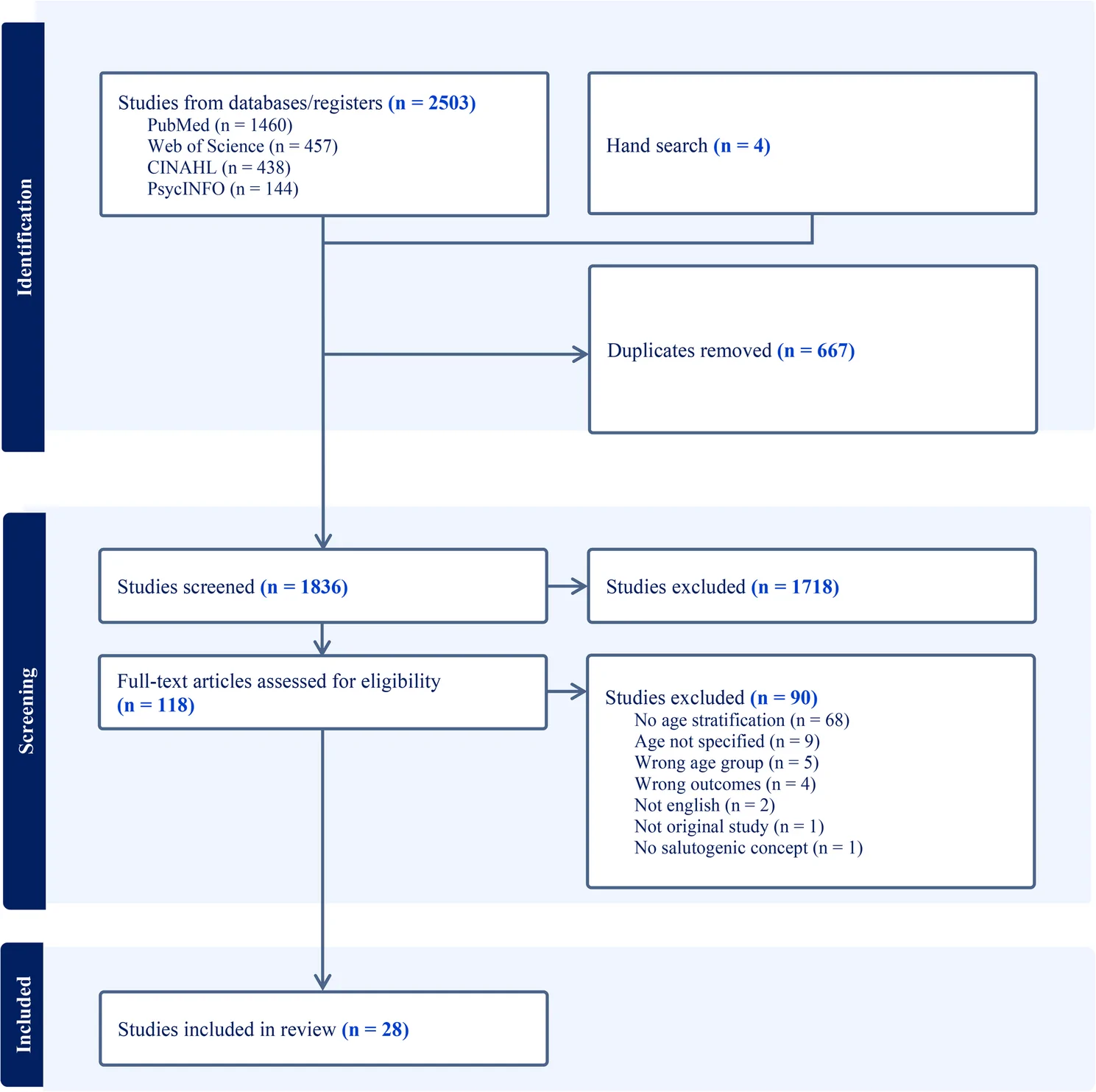
PRISMA flow chart

The initial search of the electronic databases yielded 2,503 potential articles. After removing duplicates, 1,836 titles and abstracts were screened, resulting in 118 studies for full text review. Of these, 90 were excluded for not meeting the inclusion criteria. An additional 4 studies were identified through hand searching. In total, 28 studies met the inclusion criteria for this scoping review. For details, see PRISMA flow chart (Fig. 1).

### Study characteristics

Of the 28 studies included in this review, 20 were cross-sectional [31–50], five were cohort studies [51–55], two were follow-up studies [56, 57], and one was a clinical pilot study [58]. The studies were published between 1996 and 2025 and conducted across various regions: Europe (*n* = 6) [35, 49, 50, 52, 55, 58], Asia (*n* = 8) [31, 32, 37, 39, 40, 44, 46, 53], North America (*n* = 3) [38, 41, 47], South America (*n* = 5) [34, 36, 42, 43, 45], and Oceania (*n* = 6) [33, 48, 51, 54, 56, 57]. The most frequently represented countries were Australia (*n* = 5), India (*n* = 5), Brazil (*n* = 4), Japan (*n* = 3), and Sweden (*n* = 3). Sample sizes varied from 23 to 16, 999 participants, with age distributions between 18 and 59 years. The studies encompassed both general and specific populations across different settings. Table 3 summarizes the main characteristics of the included studies (Appendix 1).

### Salutogenic concepts

Twelve salutogenic concepts were identified in oral health research involving adult population. These included the following: self-efficacy (*n* = 5) [33, 35, 47, 53, 56], sense of coherence (SOC) (*n* = 5) [31, 32, 42, 46, 49], social capital (*n* = 5) [34, 36–38, 43], coping (*n* = 4) [33, 45, 48, 55], locus of control (LOC) (*n* = 4) [41, 44, 50, 52], social support (*n* = 4) [33, 47, 56, 57], optimism (*n* = 2) [55, 57], emotional intelligence (*n* = 1) [58], motivation (*n* = 1) [40], positive emotionality (*n* = 1) [54], resilience (*n* = 1) [51], and well-being (*n* = 1) [39]. Several studies included more than one of these concepts. Table 1 presents descriptions of each salutogenic concept along with the instruments used in included studies. Where several descriptions or definitions were available, one appropriate description was selected for each concept. The results of salutogenic concepts on oral health outcomes examined across multiple studies are summarized in Table 2 to illustrate overarching patterns and consistency in measurement.

**Table 1:** – Salutogenic concepts, descriptions, and instruments used in the included studies

Salutogenic concepts	Descriptions*	Instruments used in included studies
Coping	“Constantly changing cognitive and behavioral efforts to manage specific external and/or internal demands that are appraised as taxing or exceeding the resources of the person.” It emphasizes the dynamic interaction between the individual and their environment. Lazarus & Folkman (1984) [59]	The Perceived Stress Scale (PSS) (Items not specified) The General Health Questionnaire (GHQ-12) (12-item) Ways of Coping Checklist (WCCL) (16-item)
Emotional intelligence	“A subset of social intelligence that involves the ability to monitor one’s own and others’ feelings and emotions, to discriminate among them, and to use this information to guide one’s thinking and actions.” Salovey & Mayer (1990) [60]	A questionnaire as described by Cooper & Sawaf (1997) (Items not stated)
Locus of control	“The degree to which individuals believe that they can control events affecting them.” Internal locus of control: The belief that one has control over life events. External locus of control: The belief that external factors, such as luck or other people, determine what happens. Rotter (1966) [61]	The Cluster from the Dental Coping Belief Scale (DCBS) (14-item) The Occupational Stress Indicator (OSI) (Cooper et al. 1988) (Items not stated) The Multidimensional Health Locus of Control Scale (MHLCS) (18-item) The Oral Health Locus of Control instrument (OHLoC), adapted from Health Locus of Control Scale (HLOC) (9-item)
Motivation	“The process that initiates, guides, and sustains goal-directed behavior”. Miele et al. (2024) [62]	The Dental intrinsic motivation scale (DIM-s) by Syrjälä et al. (12-item)
Optimism	A style of explaining life events where people see negative events as temporary, specific, and external, and positive events as permanent, pervasive, and internal. Seligman (1990) [63]	The Life Orientation Test (12-item) Revised Life Orientation Test (LOT-r) (6-item)
Positive emotionality	The tendency to experience positive emotions like joy, interest, contentment, pride, and love. Fredrickson (2001) [64]	The Multidimensional Personality Questionnaire (177-item)
Resilience	The capacity to cope effectively with the internal stresses of their vulnerabilities (labile patterns of autonomic reactivity, developmental imbalances, unusual sensitivities) and external stresses (illness, major losses, and dissolution of the family). Werner (1982) [65]	The Resilience construct in instrument “Strong Souls” (9-item)
Self-efficacy	“The belief in one’s capabilities to organize and execute the courses of action required to manage prospective situations.” Bandura (1997) [66]	Self-efficacy scale for self-care (SESS) (15-item) The Perceived Health Competence Scale (PHCS) (8-item) The General Self-Efficacy (GSE) instrument (10-item) The Multidimensional Home Environment Scale (MHES) (items not stated)
Sense of Coherence	The extent to which individuals perceive life as comprehensible, manageable, and meaningful. Antonovsky (1987) [4]	The Sense of Coherence Scale, SOC (13-item)
Social capital	The networks, norms, and trust that enable people to act together more effectively to pursue shared objectives. Putnam (1995) [67]	A global self-rated item (3-item) The Canadian Community Health Survey (CCHS) questionnaire (Items not specified) The Integrated Questionnaire for the Measurement of Social Capital (SC-IQ) (3-item) Self-designed questionnaire
Social support	The perception or experience that one is cared for, has assistance available from other people, and is part of a supportive social network. House (1981) [68]	The Multidimensional Scale of Perceived Social Support (MSPSS) (12-item) The Multidimensional Home Environment Scale (MHES) (items not stated)
Well-being	A state in which every individual realizes his or her own potential, can cope with the normal stresses of life, can work productively and fruitfully, and is able to contribute to his or her community. WHO (2001) [69]	The General Health Questionnaire (GHQ-12) (12-item)

* Concept definitions as chosen by the authors in this review

**Table 2:** Summary of studies examining salutogenic concepts and oral health-related outcomes

Salutogenic concept	Studies (*n*)	Objective outcomes	Subjective outcomes	Consistency of findings	Consistency of measurement
↑ Self-efficacy	5	↓ caries, ↓ periodontal progression	↓ self-reported deterioration, ↓ oral health impact	Beneficial association in 5/5 studies (mixed across outcomes)	4 different instruments used (GSE, SESS, PHCS, MHES)
↑ Sense of coherence	5	Mixed findings regarding dental caries and periodontitis	↑ self-reported oral health	Beneficial association in 5/5 studies (mixed across outcomes)	SOC-13 used in 5/5 studies
↑ Social capital	5	↓ caries	↑ self-reported oral health	Beneficial association in 5/5 studies (mixed across outcomes)	2 different instruments used (SC-IQ, CCHS) as well as study-specific measures
↑ Coping	4	↓ periodontitis, ↓ tooth loss	No significant associations with self-reported oral health	Beneficial association in 2/4 studies; no association in 2/4 studies	3 different instruments used (PSS, GHQ, WCCL). PSS used in 2/4 studies.
↑ Locus of control	4	↓ caries, ↓ periodontitis	Mixed findings regarding self-perceived oral health	Beneficial associations in 4/4 studies (mixed across outcomes)	4 different instruments used (DCBS, OSI, MHLCS, OHLoC)
↑ Social support	4	↓ tooth loss, ↓ caries	Mixed findings regarding self-perceived oral health and oral health-related quality of life	Beneficial associations in 3/4 studies; no association in 1/4 studies	2 different instruments used (MSPSS, MHES). MSPSS used in 3/4 studies

Mixed = combination of significant and non-significant findings

↑ = increased/improved ↓ = decreased

### Self-efficacy

Of the five included studies, three were cross-sectional [33, 35, 47], one follow-up study [56], and one was a cohort study [53]. Self-efficacy was studied across both general and specific populations, including younger adults, students, and low-income women using various measurement instruments (Table 3 in Appendix).

#### Periodontal disease and dental caries

One study conducted among Japanese students found that low self-efficacy in self-care was significantly associated with the progression of periodontal disease (adjusted OR = 1.516; 95% CI: 1.010–2.275; *p* < 0.05) [53]. Additionally, among low-income women in the United States, subscales measuring self-efficacy for eating healthy and oral hygiene practices were negatively associated with dental caries (adjusted β = −0.514 and adjusted β =–0.280 respectively; *p* < 0.05) [47].

#### Self-reported oral health

Among Australian adults, health-related self-efficacy, representing psychosocial resilience, was associated with a lower prevalence of self-reported worsening in oral health (adjusted PR = 0.75; 95% CI 0.57–0.99; *p* < 0.05) [56], as well as with reduced oral health impact, i.e., improved oral health-related quality of life (adjusted ß = − 3.3; *p* < 0.05) [33]. Among Swedish adolescents, a high general self-efficacy score was significantly associated with the feeling of taking good care of one’s teeth (adjusted OR = 2.20; 95% CI: 1.40–3.44; *p* < 0.001), but not with subjective oral health [35].

### Sence of coherence

All studies that examined sense of coherence (SOC) were cross-sectional [31, 32, 42, 46, 49]. SOC was studied across both general and specific populations: bus drivers, students, young males in a military setting, and middle-aged women. All studies employed the SOC 13-item scale (SOC-13) as the measurement instrument (Table 3 in Appendix).

#### Periodontal disease, dental caries and missing teeth

The studies reviewed revealed inconsistent associations between SOC and both periodontal disease and dental caries. In a study involving 600 Indian bus drivers, no significant correlations were found between SOC and the Community Periodontal Index (CPI) or loss of attachment (LOA) [31]. Conversely, a study of Indian young adults aged 19–23 years, reported a significant negative correlation between SOC and CPI (crude *r* = − 0.094; *p* < 0.05), suggesting that higher SOC scores were correlated with better periodontal health. However, no significant correlation was found between LOA and SOC in this population [32]. A third study, involving Indian adults aged 35–44 years found SOC to be negatively correlated with both CPI (crude *r* = − 0.306) and LOA (crude *r* = − 0.277), with both correlations being statistically significant (*p* < 0.05), thus indicating better periodontal status among individuals with higher SOC scores [46]. Regarding dental caries, a nonsignificant negative correlation was observed among the bus drivers [31]. Similar findings were reported in a study from India involving young adults [32], and in Brazil among 507 young military males [42]. In both studies, no significant association was found between SOC and the mean Decayed, Missing, and Filled Teeth (DMFT) score. However, in the Brazilian study, a higher SOC was significantly associated with a lower number of active caries lesions (adjusted RR = 0.85; 95% CI: 0.74–0.98).

In a Swedish study of middle-aged women, after adjusting for socioeconomic status (SES), the number of teeth was significantly related to SOC. Specifically, a greater number of missing teeth was linked to lower SOC levels among 50-year-olds (adjusted OR = 1.02; 95% CI: 1.01–1.04, *p* = 0.018). However, no statistically significant associations were found between SOC and either caries or filled surfaces [49].

#### Self-reported oral health

Two cross-sectional studies – one from India and one from Sweden – examined the relationship between self-reported oral health and SOC. The Indian study found that individuals reporting good perceived oral health had higher levels of SOC (crude *r* = − 0.303, *p* < 0.05) [46]. In contrast, the Swedish study, which included women aged 38 and 50, revealed a statistically significant association between SOC and self-reported oral health in both age groups (adjusted OR 1.03; 95% CI: 1.01–1.04, *p* = 0.006) [49].

### Social capital

Social capital was studied in five cross-sectional studies [34, 36–38, 43], primarily involving general population samples. However, one study focusing on college students [37] assessed social capital in more specific domains: family, neighborhood, and school. As shown in Tables 3 in Appendix and Table 1, the instruments used to measure social capital varied.

#### Dental caries

Two cross-sectional studies from Brazil [34, 43] examined the relationship between social capital and dental caries in adults aged 35–44 years. One study found that low social capital was associated with a higher DMFT index (adjusted OR 1.25; 95% CI: 1.14–1.37) [43]. The other study examined caries severity in relation to social capital across multiple levels – individual, social structure, and social context – within the adult population. A greater prevalence of higher caries severity was found in relation to social capital and reported as “neighbourhood did not come together in the previous years to petition political leaders for benefits” (adjusted PR = 1.16; 95% CI 1.05–1.28) and “those who are unable to make decisions (without empowerment)” (adjusted PR = 1.12; 95% CI: 1.01–1.24) [34].

#### Self-reported oral health

Three cross-sectional studies explored the associations between social capital and self-perceived oral health (SPOH). One study, conducted among the general population aged 35–44 years in Brazil examined SPOH in relation to different aspects of social capital. The findings indicated that individuals with low social capital were more likely to report a higher rate of poor SPOH (adjusted PR = 1.20; 95% CI: 1.10–1.31 [36]. Another study conducted in Japan among colleague students examined social capital across various settings, including family, neighborhood, and schools. Poor SPOH was significantly associated with lower levels of social capital, specifically neighborhood trust (adjusted OR = 2.22; 95% CI: 1.40–3.54) and vertical trust within schools (adjusted OR = 1.71; 95% CI: 1.05–2.80). Better SPOH was associated with lower informal social control (adjusted OR = 0.54; 95% CI: 0.34–0.85), while family social capital showed no significant association with SPOH [37]. In a Canadian population-based study, social capital was assessed through indicators such as sense of belonging to the local community and living or family arrangements. Younger adults (aged 30–44 years) were found to have a higher risk of inadequate self-perceived oral health when experiencing high levels of perceived life stress combined with a low sense of belonging (adjusted PR = 1.57; 95% CI: 1.46–1.69), or when living alone (adjusted PR = 1.33; 95% CI: 1.21–1.47). Similarly, middle-aged adults (45–59 years) showed a higher risk of inadequate self-perceived oral health under the same conditions – high perceived life stress and low sense of belonging (adjusted PR = 1.79; 95% CI: 1.66–1.94), or living alone (adjusted PR = 1.54; 95% CI: 1.41–1.68) [38].

### Coping

Coping was studied in four cross-sectional studies [33, 45, 48, 55], as shown in Table 3 in Appendix. The concept of coping was explored in both general populations and specific groups: students at a police academy. Across these studies, three different instruments were used to assess coping, as detailed in Table 3 in Appendix and Table 1.

#### Periodontal disease and missing teeth

A study conducted among students in Trinidad and Tobago found that an inability to cope with stress was a statistically significant predicator of periodontitis compared to gingivitis (adjusted OR = 1.45; 95% CI: 1.15–1.82) [45]. In another study among Australian adults aged 43–57 years, a negative relationship was observed between coping, measured through perceived stress scores, and the retention of fewer than 20 teeth (adjusted OR = 1.4; 95% CI: 1.3–1.5) [48].

#### Self-reported oral health

Two cross-sectional studies investigated the relationship between coping and oral health outcomes. One study examined active coping in relation to self-reported dental health among 31-year-old men and women in Finland [55], while the other explored coping in relation to oral health-related quality of life among adults aged 45–54 years in Australia [33]. Neither study found a significant association between coping and self-reported oral health.

### Locus of control

Locus of control (LoC) was studied in three cross-sectional studies [41, 44, 50] and one cohort-study [52], as shown in Table 3 in Appendix. The concept was explored in both general populations and specific groups, including sickle-cells disease, employed adults, and young adults. Across these studies, four different instruments were used to assess LoC (Table 1).

#### Periodontitis disease and dental caries

A study comparing two African American groups, with and without sickle cell disease (SCD), found that individuals in the highest quartile of dental external locus of control had significantly more untreated dental decay compared to those in the lowest quartile (adjusted 2.58-fold increase, 95% CI: 1.05–6.34; and adjusted 3.00-fold increase, 95% CI: 1.38–6.49, respectively) [41]. In another study, examining occupational stress, LoC was included in a regression model that explained 65% of the variance in a periodontal attachment loss [52]. Moreover, a positive correlation was observed between LoC and both the CPI (crude *r* = 0.1219) and LOA (crude *r* = 0.1041) [44].

#### Self-reported oral health

One study conducted among Swedish 19-year-olds examined the relationship between Oral Health Locus of Control (OHLoC) and self-perceived oral health. A higher degree of internal OHLoC was associated with a lower probability of reporting poor self-perceived oral health (adjusted OR = 0.84, 95% CI: 0.73–0.96). On the other hand, a higher degree of chance OHLoC was linked to an increased risk of perceiving one’s oral health as poor (adjusted OR = 1.10, 95% CI: 1.00–1.22). However, this association was modified by health behaviors. No significant association was found between the external OHLoC subscale displayed and self-perceived oral health [50].

### Social support

Among the four included studies, social support was investigated in two cross-sectional studies [33, 47] and two follow-up studies [56, 57], as shown in Table 3 in Appendix. Social support was examined in both general populations and specific groups, such as low-income women, using two different assessment instruments (Table 3 in Appendix and Table 1).

#### Dental caries

Statistically significant relationships between social support and oral health were reported in two studies. In a US study among low-income women aged 18–50 [47], social support was significantly associated with dental caries, even after adjusting for socioeconomic factors (adjusted ß = 0.171; *p* < 0.05). A 17-year follow-up study conducted in Australia among individuals aged 30 years found a negative association between social support and both the number of missing teeth (adjusted ß = -1.0) and caries experience (adjusted ß = -1.5) [57].

#### Self-reported oral health

A cross-sectional study conducted in Australia among a general population aged 45–54 years found a statistically significant association between higher levels of social support and lower oral health impact (adjusted ß = -0.1; *p* < 0.05) [33]. However, a 2-year follow-up study based on the same population as the Australian cross-section study, did not find a significant association between social support and self-reported changes in oral health [54].

### Other concepts

In addition to the concepts described, several other concepts were found in this review, though only appearing in a limited number of studies. These included emotional intelligence [58], motivation [40], optimism [55, 57], positive emotionality [54], resilience [51], and well-being [39]. These concepts were examined through various study designs: one follow-up study [57], four cross-sectional studies [39, 40, 51, 54], one cohort study [55], and one clinical pilot study [58].

Different domains of emotional intelligence (EI) were examined in a study conducted in the UK among patients with chronical periodontitis (58). Changes in bleeding were positively correlated with several EI domains, including intentionality (crude *r* = 0.358, *p* = 0.057), resilience (crude *r* = 0.519, *p* = 0.004), constructive discontent (crude *r* = 0.542, *p* = 0.002), personal power (crude *r* = 0.374, *p* = 0.045), and intuition (crude *r* = 0.433, *p* = 0.019). No other EI domains showed statistically significant correlations with changes in bleeding.

The concept of motivation was explored in an Indian study among engineering college students, which assessed dental intrinsic motivation in relation to caries status (DMFT). Although both the decayed component and the overall DMFT score showed a positive correlation, the association was not statistically significant [40].

The concept of optimism was investigated in two studies. A Finnish study involving 31-year-olds found that the higher optimism was associated with a lower prevalence of self-reported caries (adjusted PPR = 0.73, 95% CI 0.65–0.82) and gingival bleeding (adjusted PPR = 0.62, 95% CI: 0.65–0.82) [55]. In an Australian study of 30-year-olds, optimism was significantly associated with better oral health, showing negative associations with both missing teeth (adjusted β = −0.987, *p* < 0.01) and caries experience (adjusted β = −1.481, *p* < 0.05) for high social support with high optimism compared to low social support with low optimism [57].

A study conducted in New Zealand among 32-year-olds examined the role of positive emotionality in relation to oral health outcomes but found no statistical significant associations [54]. In another study involving Aboriginal adults, oral health-related factors – including DMFT, attachment loss, and oral health impact – were assessed using resilience as a measure of social and emotional well-being. Resilience was significantly associated with having one or more filled teeth (adjusted B = 1.71; 95% CI: 0.66–2.77), while no other significant associations were observed [51].

In a study among Australian civil service officers, psychological well-being was not significantly associated with dental caries, periodontal status, or the number of missing teeth. However, subjects who rated their oral health as poor reported poorer psychological well-being compared to those who rated their oral health as excellent or good, although there was only a statistically significant difference among females (*p* = 0.004) [39].

## Discussion

To the best of the authors’ knowledge, this is the first review to systematically identify and map existing scientific research on salutogenic concepts as oral health resources among adults, applied to dental caries, periodontitis, and self-reported oral health. The review provides a novel and comprehensive overview of how the salutogenic theory has been utilized within oral health research to date. Based on the salutogenic umbrella framework [14], the review identified more than half of the 21 concepts. Starting with the “locus of control” in 1996 [52], a notable increase in the use of salutogenic concepts has been observed since the mid-2010s, thus reflecting a growing worldwide interest in this theoretical approach.

The results suggest that positive psychosocial resources, when viewed through the lens of the salutogenic theory, are associated with more favorable oral health outcomes across diverse adult populations. This can be applied to outcomes including dental caries, periodontitis, and self-reported oral health. Notably, particular emphasis has been placed on key concepts such as self-efficacy, SOC, and social capital.

The reviewed studies examining self-efficacy in relation to oral health suggest that it may serve as an important psychosocial factor in oral health [33, 35, 47, 53, 56]. These findings align with theoretical perspectives that view self-efficacy as a key component in health-related behavior change and maintenance [66]. They also align with previous research showing that higher levels of self-efficacy are associated with better oral health outcomes, including improved gingival health in older adults and more frequent tooth brushing among adolescents [21, 70].

Similarly, studies examining the association between SOC and oral health indicate that a higher SOC is associated with more favorable oral health outcomes, both in terms of clinical indicators and self-reported oral health. However, the strength and significance of these associations varied between studies [31, 32, 42, 46, 49]. Broader evidence supports these findings, indicating that individuals with higher SOC tend to have better oral clinical status [18, 23, 25, 26], engage more frequently in positive oral health behaviors, such as tooth brushing and preventive dental visits [18, 71], and report higher oral health-related quality of life [19]. Collectively, these results highlight SOC as an important psychosocial factor in the promotion and maintenance of oral health.

Notably, all reviewed studies investigating the relationship between social capital and oral health outcomes adopted a pathogenic perspective. The studies reported that lower levels of social capital were associated with poorer self-reported oral health and greater caries experience and severity [34, 36–38, 43]. From a salutogenic perspective, however, it is plausible that higher social capital may be associated with improved oral health outcomes. This is consistent with previous findings in children and adolescents, which has shown that both contextual and individual social capital are positively associated with both clinical indicators and self-reported measures of oral health [72].

Higher levels of optimism among young adults have been consistently linked to improved oral health outcomes [55, 57. Moreover, either high optimism or social support may buffer against the negative impact of dental problems [57]. These findings indicate that both optimism and social support is associated with more favorable oral health outcomes. The results are in line with previous research that identifies these constructs as psychological resources and reliable predictors of positive physical health outcomes across diverse populations and health conditions [73–75].

Among the studies included in this review, under-explored concepts – such as emotional intelligence, resilience, and optimism – have yielded promising yet mixed results. These findings suggest that such salutogenic resources may have potential benefits for oral health outcomes. However, further research is needed to better understand their impact within adult populations.

Other studies have explored these concepts in adult populations, focusing on outcomes such as dental caries, periodontitis, and self-reported oral health. Examples include research on sense of coherence [18, 76], intention and self-esteem [77], interdisciplinary [78], attachment [79], humor [80], empowerment [81], and oral health literacy [82]. However, these were excluded from the current review due to insufficient age-related reporting. Complimentary qualitative research has also highlighted the relevance of salutogenic resources in promoting oral health among adults [83–85]. These studies collectively show an increased international interest in applying the salutogenic model to adult oral health. This trend indicates a paradigm shift in oral health research – from a pathogenic focus toward a health-promotive approach [9, 10] – and reflects the prioritization of oral health as a critical public health concern worldwide [2, 27].

The salutogenic concepts examined in the included studies varied in their conceptual clarity. While several were well-defined, others lacked precision, and several demonstrated conceptual overlap. For example, self-efficacy was used to represent psychosocial resilience in one study [56], whereas resilience was measured as social and emotional well-being in another [51], and as emotional intelligence in a third [58]. Additionally, certain concepts included subdimensions, for example, social capital was subdivided into family, neighborhood, and school-based social capital [37]. While several salutogenic concepts are conceptually related, they represent distinct theoretical constructs operating at different levels. In this review, efforts were made to distinguish these constructs based on their theoretical origins. Conceptual analysis plays a critical role in clarifying the meaning of central concepts within both theory and practice [86]. To advance evidence-based knowledge in salutogenic oral health research, we recommend the use of well-defined and coherent concepts.

Methodologically, the studies utilized a combination of validated and self-designed instruments, reflecting varying degrees of psychometric rigor. The use of well-established scales, such as SOC-13, GHQ-12, and MHLC, enhances the reliability and facilitates comparability across studies. However, reliance on self-developed questionnaires or instruments that lack detailed information, such as the number of items, reduces methodological transparency and hinders standardization [87]. This is particularly concerning given the theoretical overlaps of the salutogenic concepts [88]. Furthermore, the utilized instruments varied substantially in length, ranging from ultra-brief measures such the 3-item SC-IQ to extensive tools such as the 177-item Multidimensional Personality Questionnaire. While shorter instruments tend to lessen respondent burden and improve completion rates, longer tools may capture more detailed and multidimensional assessments of the constructs under investigation. Ultimately, the choice of instrument should primarily be guided by its content relevance and psychometric quality, rather than its length alone [89]. This conceptual complexity poses challenges for comparing results and drawing definitive conclusions. This highlights the need for further research that enhances validity, reliability, and cross-study comparability in the application of salutogenic concepts. Moreover, development of robust salutogenic measurement tools, both quantitative and qualitative, is recommended as such tools are essential for informing and guiding effective health promotion interventions [8]. Future research could benefit from the use of validated, context-relevant instruments with clearly documented properties to improve study quality and facilitate cross-study comparability.

While this review identified several applications of salutogenic concepts within oral health sciences, in relation to dental caries, periodontitis, and self-reported oral health, significant knowledge gaps remain. Empirical research – particularly longitudinal and intervention studies – continues to be limited regarding the causal pathways between salutogenic constructs and oral health outcomes among adults. Furthermore, conceptual uncertainty persists not only in relation to the salutogenic theory itself but also concerning health promotion, which is often considered as its practical counterpart. Advancing research in this field therefore requires a clearer theoretical understanding of both salutogenesis and health promotion as foundational frameworks [5, 90]. However, recent developments in salutogenesis research suggest a holistic biopsychosocial health model. This model encompasses the full spectrum of human health experiences, including negative pathogenic stressors, coping strategies, and positive salutogenic recourses, making it universally applicable across contexts [8]. This perspective also underscores the importance of distinguishing and integrating the concepts of prevention and health promotion, which, while conceptually distinct, function as complementary and concurrent processes within health-related interventions [86].

This study, through a salutogenic lens on oral health, contributes meaningfully to the field of oral health promotion research. Health promotion is defined as “the process of enabling people to increase control over, and to improve, their health” [7]. From a salutogenic perspective, life challenges are seen as inherent and potentially beneficial, as coping with them and mobilizing health resources can contribute to learning, empowerment, and strengthened autonomy [3, 11]. Focusing on salutogenic resources in oral health interventions may therefore support individuals in progressing towards better health, particularly in response to life stressors. This approach is consistent with the Ottawa Charter, which highlights the role of social and personal resources in health promotion [7], and supports the view that such resources represent key outcomes of health-promotive efforts [91].

### Strengths and limitations

This scoping review has systematically mapped the field of salutogenic oral health, identifying key concepts that clarify existing knowledge and highlight gaps for future research. This constitutes a key strength, positioning the study as a valuable complement to existing research within the area of salutogenesis. Notably, the review builds upon the salutogenic theory, and the search string adhered to the salutogenic umbrella framework. However, certain limitations should be acknowledged. The search string did not include salutogenic concepts and factors such as health literacy, motivation, or self-esteem, which may have excluded relevant studies. Further, while the inclusion of QoL in the search string generated numerous articles where QoL was treated as an outcome measure, no studies were identified that explored QoL as a salutogenic resource. Also, studies published after February 2025 were not included. An updated search was conducted only in PubMed rather than across all databases included in the original search, as PubMed was considered the database with the broadest coverage of the research area. As a result, potentially relevant studies, particularly those indexed exclusively in other databases, may have been omitted from this scoping review. Further, in accordance with the eligibility criteria, 77 studies were excluded due to either not reporting the age range of participants or not stratifying results by age group. These limitations may have introduced selection bias.

Furthermore, a notable pattern among the included studies is the predominant use of cross-sectional designs. While such designs are valuable for identifying associations, they inherently limit the ability to draw causal inferences [92]. Moreover, differences across study designs, populations, and research instruments further limit the potential for generalization of results across broader contexts. Information regarding validation of measurement instruments was inconsistently reported across studies and could therefore not be systematically assessed. Methodological limitations have also been highlighted in a previous systematic review and meta-analysis examining protective psychosocial factors, based on the salutogenic theory, in relation to dental caries among children and adolescents [22]. Nonetheless, the inclusion of cohort and follow-up studies in the current review provides valuable insight into potential longitudinal effects, particularly regarding optimism and social support.

### Authors’ recommendations

1. Use of well-defined and coherent concepts to advance evidence-based knowledge in salutogenic oral health research
2. Consider the application of salutogenesis as the theoretical basis for interventions at all levels and incorporate salutogenic resources as a major part of health promotion
3. Perform longitudinal studies, preferable Randomized Controlled Trials (RCTs), to determine and evaluate the causality and effect of salutogenic resources on oral health.

## Conclusion

This review systematically identified and mapped the existing scientific literature on salutogenic concepts as oral health resources in adult populations. The findings suggest a growing international interest in applying salutogenic perspectives to oral health, thus reflecting a potential paradigm shift from a predominantly pathogenic focus toward a more health-promotive approach. This shift holds significant implications for the development of oral health-promotive strategies aimed at fostering sustainable improvements. Salutogenic concepts were frequently associated with favorable oral health outcomes and offer a valuable complement to traditional preventive strategies in oral health research and practice. However, to advance evidence-based knowledge in this area, the field requires the development of clearly defined concepts and the implementation of methodologically robust studies.

## Supplementary Information

Supplementary Material 1.

Supplementary Material 2.

## Data Availability

No datasets were generated or analysed during the current study.

## Appendix

**Table 3:** Salutogenic concepts, descriptions, and instruments used in the included studies

Author, year, country	Sample, age, total number	Study design	Setting	Aim	Outcome measures	Salutogenic concepts	Instruments	Concluding results
Ahmed et al. (2018) [31], India	Bus drivers, 35–55 yrs, 600	Cross sectional study	Workplace	To assess the SOC and its impact on oral hygiene behavior and oral health status among bus drivers	DT DMFT CPI LOA	SOC	SOC-13 (13-item)	Higher SOC was non-significantly correlated with better oral health outcomes
Anu et al. (2025) [32], India	Young adults, 19–23 yrs, 500	Cross sectional study	Educational institutions	To assess the impact of SOC with Oral Hygiene, dental caries and periodontal status among young adults in Chennai, Tamil Nadu	DMFT CPI LOA	SOC	SOC-13 (13-item)	SOC had a negative correlation with Loss of attachment. There was no significant relation between SOC and other oral health outcomes.
Brennan et al. (2019) [56], Australia	Adults, 45–54 yrs, 986	2-year follow-up	Household	To examine changes in health associated with perceived stress, social support, and self-efficacy, controlling for sociodemographic characteristics and health behavior	Self-reported changes in oral health	Social support Health self-efficacy	Multidimensional scale of perceived social support (12-item) Perceived Health Competence Scale, 8-item	Social support was not significantly associated with self-reported changes in oral health. Participants who perceived higher health competence were less likely to report worsening in oral health.
Brennan et al. (2021) [33], Australia	Adults, 45–54 yrs, 986	Cross sectional study	Household	To investigate oral health impacts by subjective social status (SSS) and psychosocial predictors stratified by subjective social status	OHIP-14	Social support Health self-efficacy Coping	Multidimensional scale of perceived social support (12-item) Perceived Health Competence Scale, 8-item Perceived Stress Scale (Items not specified)	More social support and health self-efficacy were associated with lower oral health impact. Coping was not significantly associated with oral health impact
Brennan & Spencer (2012) [57], Australia	Young adults, 30 yrs, 1859	17-year follow-up study	Household, dental clinic	To assess associations of social support and optimism on oral health	DMFT OHIP	Social support Optimism	Multidimensional Scale of Perceived Social Support (12-item) the Life Orientation Test (12-item)	Social support and optimism had negative associations with missing teeth and caries experience for high support/high optimism compared to low support/low optimism. Impact of dental problems showed buffering of high support when optimism was low, and high optimism when support was low.
Costa et al. (2012) [34], Brazil	Adults, 35–44 yrs, 1150	Cross sectional study	Household	To assess the association between caries severity in adults aged 35 to 44 years and the characteristics of this population with respect to the different levels at which the determinants of caries operate (individual, social structure and social context levels)	DMFT	Social capital	Self-designed questionnaire	Two of five dimensions of social capital (Community petitioned authorities for neighborhood benefits and capable of making decisions) was negatively associated with caries severity
Ericsson et al. (2016) [35], Sweden	Adolescents, 19 yrs, 506	Cross sectional study	Dental clinic	To test the hypothesis that certain individual, environmental and lifestyle factors are positively associated with beneficial health investment behaviors and oral/periodontal health among adolescents	Self-reported oral health	General self-efficacy	General Self-Efficacy (GSE) instrument (10-item)	General self-efficacy was not associated with self-reported oral health, but was significantly positively associated with the feeling of taking good care of the teeth
Fagundes et al. (2021) [36], Brazil	Adults, 35–44 yrs, 5815	Cross sectional study	Household	To determine the factors associated with self-perceived oral health (SPOH) in different age groups	Self-perceived oral health (SPOH)	Social capital	A global self-rated item (3-item)	Lower social capital was associated with higher rates of poor self-perceived oral health
Furuta et al. (2012) [37], Japan	College students, 18–19 yrs, 967	Cross sectional study	Educational institutions	To examine the associations of social capital in three settings (families, neighborhoods, and schools) with self-rated oral health among a sample of college students in Japan	Self-rated oral health	Family social capital Neighborhood social capital School social capital	Not stated (1-item) Not stated Not stated	Family social capital was not significantly associated with self-rated oral health. Neighborhood social capital (higher neighborhood trust and lower informal social control, respectively) was significantly associated with better self-rated oral health Lower level of vertical trust in school, but not horizontal trust in school was significantly associated with poor self-rated oral health
Gamboa et al. (2005) [58], UK	Patients with chronic periodontitis, 35–58 yrs, 29	Clinical pilot study	Dental clinic	To investigate the relationship between different domains of EI, and initial response to a simplified non-surgical treatment protocol in 29 patients with chronic periodontitis	Change in bleeding	Emotional intelligence	A questionnaire as described by Cooper & Sawaf (1997) (Items not stated)	Intentionality, resilience, constructive discontent, and intuition, but not other emotional intelligence domains, were positively correlated to changes in bleeding.
Hensel and Gomaa (2023) [38], Canada	Adults, 30–59 yrs, 16,999	Cross sectional study	Household	To assess the extent of the association of psychosocial stress with oral health in an Ontario population stratified by age groups, and whether any association is modified by indicators of social and economic capital	Perceived oral health, bleeding gums	Social capital	The Canadian Community Health Survey (CCHS) questionnaire (Items not specified)	Low sense of belonging and high perceived life stress, as well as living alone with higher perceived life stress was associated with higher risk of inadequate oral health.
Ide et al. (2006) [39], Japan	Civil service officers, 20–59 yrs, 1381	Cross sectional study	Workplace	To examine the association between psychological well-being and oral conditions of Japanese worker	CPITN Self-rated oral health DT MT	Psychological well-being	General Health Questionnaire (GHQ-12)	Psychological well-being was not associated significantly with oral health outcomes.
Jamieson et al. (2011) [51], Australia	Aboriginal, 18 yrs, 336	Cohort study	Household, dental clinic	To investigate associations between oral health-related factors and social and emotional well-being in a birth cohort of young Aboriginal adults residing in the northern region of Australia’s Northern Territory	DMFT Attachment loss Oral health impact	Resilience	Resilience construct in instrument “Strong Souls” (9-item)	Resilience was significantly associated with having one or more filled teeth, but not with any other variable.
Kumar et al. (2019) [40], India	Engineering college students, 18–20 yrs, 393	Cross sectional study	Educational institutions	To determine the effect of motivation on oral hygiene and caries status among young adults in Hyderabad city	DMFT	Dental intrinsic motivation	Dental intrinsic motivation scale (DIM-s) by Syrjälä et al. (12-item)	Dental intrinsic motivation was not significantly associated with DMFT.
Laurence et al. (2006) [41], USA	African American with/without SCD, 18–59 yrs, 205	Cross sectional study	Hospital, dental clinic	To investigate the association between self-perceived loss of control as measured by dental external locus of control summary scores, with the amount of untreated dental decay in African American adults with sickle cell disease (SCD) and African Americans adults without SCD	DMFS	Dental external locus of control	cluster from the Dental Coping Belief Scale (DCBS) (14-item)	Higher level of dental external locus of control was associated with increased untreated decay.
Linden et al. (1996) [52], Northern Ireland	Employed regular dental attenders, 20–50 yrs, 23	Cohort study	Dental clinic	To examine the association between occupational stress and the progression of periodontitis in employed adults	BOP, probing depth and clinical attachment level	Locus of control	The Occupational Stress Indicator (Cooper et al. 1988) (items not stated)	Locus of control was significant associated with progressive periodontitis, those who felt less in control of their environment tended to have greater change in attachment.
Lock et al. (2024) [42], Brazil	Male adolescents, 18–19 yrs, 507	Cross sectional study	Military base	to assess the association between SoC and caries activity and caries experience among 18- to 19-year-old male adolescents from southern Brazil	DMFT	SOC	SOC-13 (13-item)	A high SoC was significantly associated with a lower number of active lesions, but not with DMFT.
Mizutani et al. (2015) [53], Japan	Students, 18–30 yrs, 533	Cohort study	Educational institutions	To investigate whether self-efficacy scale for self-care (SESS) was related to changes in oral health behaviors and progression of periodontal disease in Japanese university students	CPI, BOP, PPD	Self-efficacy	Self-efficacy scale for self-care (SESS) (15-item)	Low self-efficacy for brushing of the teeth was significantly associated with progression of periodontal disease, but not self-efficacy for dentist consultations and dietary habits.
Nogueira et al. (2019) [43], Brazil	Adults, 35–44 yrs, 6051	Cross sectional study	Household	To investigate the experience of caries related to social capital and associated factors in adults in large-scale population-based study	DMFT	Social capital	questions from the SB SP 2015 form, extracted from Integrated Questionnaire for the Measurement of Social Capital (SC-IQ) (3-item)	Low social capital was significantly associated with higher DMFT.
Padmaja et al. (2018) [44], India	IT professionals, 18–50 yrs, 989	Cross sectional study	NR	To assess the relationship between health locus of control and oral health status among Information Technology (IT) professionals in Hyderabad, India.	CPI LOA	Health Locus of Control	Multidimensional Health Locus of Control Scale (MHLCS) (18-item)	External Locus of Control was positively correlated with CPI and LOA respectively.
Ramlogan et al. (2020) [45], Trinidad and Tobago	Police academy recruits, 19–35 yrs, 85	Cross sectional study	Educational institutions	To investigate self-reported stress level and coping ability as well as mental status (anxiety and depression) via the 12-item General Health Questionnaire (GHQ-12) questionnaire and periodontal status among police academy recruits during their 8 months of training	Periodontal status	Coping	General Health Questionnaire (GHQ-12) (12-item)	Inability to cope with stress was a significant predictor for periodontitis.
Reddy et al. (2016) [46], India	Adults, 35–44 yrs, 780	Cross sectional study	Educational institutions	to evaluate the effect of SOC; hence, an attempt has been made in the present study to correlate the SOC with oral health behaviors, socioeconomic status, and periodontal status among 35–44 years adults visiting Panineeya Mahavidyalaya Institute of Dental Sciences and Research Centre, Hyderabad	CPI LOA Perceived oral health	SOC	SOC-13 (13-item)	CPI, LOA and perceived oral health were significantly and negatively correlated with SOC
Sachdev et al. (2022) [47], USA	Low-Income Women, 18–50 yrs, 220	Cross sectional study	Local communities	To investigate relationships between dental nutrition knowledge, socioecological factors, and prevalence of dental caries in low-income women.	DMFT	Social support Self-efficacy for eating healthy Self-efficacy for healthy oral hygiene habits	Multidimensional Home Environment Scale (items not stated) Multidimensional Home Environment Scale (items not stated) Multidimensional Home Environment Scale (items not stated)	Higher social support, self-efficacy for eating healthy and self-efficacy for healthy oral hygiene practices were significantly related to lower dental caries levels.
Sanders et al. (2007) [48], Australia	Adults, 43–57 yrs, 2915	Cross sectional study	Household	It was hypothesized that distress and coping account, in part, for socio-economic inequality in tooth loss, and hence represent a mediator of this relationship.	Tooth loss (self-reported number of teeth)	Coping	Perceived Stress Scale (PSS) (items not reported)	Coping had a protective effect against tooth loss.
Thomson et al. (2011) [54], New Zealand	Adults, 32 yrs, 916	Cohort study	Own dental room Dedicated assessment room	To characterize the nature of the association between personality characteristics, clinical disease status and self-reported oral health in a longstanding cohort study	OHIP-14, Self-rated oral health, decayed surfaces, MT, periodontitis	Positive emotionality	the Multidimensional Personality Questionnaire (177 item)	Positive emotionality was not significantly associated with oral health outcomes.
Wennström et al. (2013) [49], Sweden	Women, 38 and 50 yrs, 493	Cross sectional study	Own dental room Dedicated assessment room	To analyze the relationship between sense of coherence (SOC), dental anxiety (DA) and oral health status among middle-aged women, measured both subjectively and objectively and adjusted for socioeconomic status (SES)	Self-reported oral health, the number of teeth, approximal caries, and the number of filled surfaces	SOC	SOC-13 (13-item)	Higher SOC levels were associated with better oral health, as estimated by objective or subjective measures.
Ylöstalo et al. (2003) [55], Finland	Adults, 31 yrs, 8690	Cohort study	Household	To investigate active coping in relation to optimism, dental health behavior and self-reported dental health among 31-yr-old men and women born in Northern Finland in 1966.	Self-reported dental health	Coping Optimism	Ways of Coping Checklist (16 item) Revised Life Orientation Test (LOT-r) (6-item)	Active coping was not related to dental health. Higher optimism was associated with less self-reported caries and gingival bleeding
Östberg and Abrahamsson (2013) [50], Sweden	Adolescents, 19 yrs, 758	Cross sectional study	Dental clinic	To test and evaluate the psychometric properties of the Oral Health Locus of Control (OHLoC) instrument and its relation to dental anxiety, self-efficacy and self-perceived oral health among Swedish adolescent	Self-perceived oral health	Oral Health Locus of Control (OHLoC)	The Oral Health Locus of Control instrument (OHLoC), adapted from Health Locus of Control Scale (HLOC) (9-item)	Higher internal OHLoC and chance OHLoC respectively was associated with poor self-perceived oral health. External OHLoC was not associated with self-perceived oral health.

*CPI* Community periodontal index, *DMFT *Decayed missing filled teeth, *DT* decayed teeth, *LOA *Loss of attachment index, *MT *Missing teeth, *NR *Not reported, *OHIP-14 *Oral health impact profile, *OHLoC *Oral health locus of control, *SOC* Sense of coherence

## Abbreviations

CPI: Community Periodontal Index
DMFT: Decayed, Missing or Filled Teeth
EI: Emotional intelligence
LOA: Loss of attachment
LOC: Locus of control
OHLoC: Oral Health Locus of Control
PCC: Population, Concept, and Context framework
PRISMA: ScR-Preferred Reporting Items for Systematic Reviews and Meta-Analyses extension for Scoping Reviews
QoL: Quality of Life
SOC: Sense of coherence
SPOH: self-perceived oral health
