# Supplementary material for: A lens on salutogenic concepts as resources for oral health among adults: a scoping review
Source: BMC Oral Health. 2026 Nov 12;26:2249. doi: 10.1186/s12903-026-10341-2 (PMC13757107; doi:10.1186/s12903-026-10341-2)
Supplement: Supplementary file 1 [file 12903_2026_10341_MOESM1_ESM.docx]

**Search string 231003**

A lens on salutogenic concepts as resources for oral health among adults: a scoping review

PubMed via the National Library of Medicine

| **No** | **Description** | **Search string** | **Hits** |
| --- | --- | --- | --- |
| #1 | P (Population) | **adult[title/abstract] OR "Adult"[Mesh]** | 8,483,603 |
| #2 | C (Concept) | **"Sense of Coherence"[Mesh] OR "Emotional Intelligence"[Mesh] OR "Self Efficacy"[Mesh] OR "Social Capital"[Mesh] OR "Resilience, Psychological"[Mesh] OR "Empowerment"[Mesh] OR "Adaptation, Psychological"[Mesh] OR "Internal-External Control"[Mesh] OR Gratitude[Title/Abstract] OR Learned resourcefulness[Title/Abstract] OR learned optimism[Title/Abstract] OR learned hopefulness[Title/Abstract] OR Sense of coherence[Title/Abstract] OR emotional intelligence[Title/Abstract] OR self-efficacy[Title/Abstract] OR cultural capital[Title/Abstract] OR Connectedness[Title/Abstract] OR Hardiness[Title/Abstract] OR social capital[Title/Abstract] OR Resilience[Title/Abstract] OR Flourishing[Title/Abstract] OR Action competence[Title/Abstract] OR Empathy[Title/Abstract] OR Empowerment[Title/Abstract] OR Will to meaning[Title/Abstract] OR Interdisciplinary[Title/Abstract] OR Humour[Title/Abstract] OR Coping[Title/Abstract] OR locus of control[Title/Abstract] OR Wellbeing[Title/Abstract] OR Ecological system theory[Title/Abstract] OR salutogenesis[Title/Abstract]** | 578,552 |
| #3 | C (Context) | **"Dental Caries"[Mesh] OR "Oral Health"[Mesh] OR "Periodontal Diseases"[Mesh] OR dental caries[Title/Abstract] OR tooth decay[Title/Abstract] OR oral health[Title/Abstract] OR periodontitis[Title/Abstract] OR periodontal disease[Title/Abstract]** | 187,618 |
| #4 | PCC | #1 AND #2 AND #3 | 1,460 |

Web of Science via Clarivate

| **No** | **Description** | **Search string** | **Hits** |
| --- | --- | --- | --- |
| #1 | P (Population) | TS=(adult) | 2,018,873 |
| #2 | C (Concept) | TS=( Gratitude OR "Learned resourcefulness" OR "learned optimism" OR "learned hopefulness" OR "Sense of coherence" OR "emotional intelligence" OR "self-efficacy" OR "cultural capital" OR Connectedness OR Hardiness OR "social capital" OR Resilience OR Flourishing OR "Action competence" OR Empathy OR Empowerment OR "Will to meaning" OR Interdisciplinary OR Humour OR Coping OR "locus of control" OR Wellbeing OR "Ecological system theory" OR salutogenesis) | 856,100 |
| #3 | C (Context) | TS=( "dental caries" OR "tooth decay" OR "oral health" OR "periodontitis" OR "Periodontal Disease") | 100,719 |
| #4 | PCC | #1 AND #2 AND #3 | 457 |

TS= Topic, Title, abstract key words

Cinahl via Ebsco

| **No** | **Description** | **Search string** | **Hits** |
| --- | --- | --- | --- |
| #1 | P (Population) | MH "Adult+" OR TI adults OR AB adults | 2,221,899 |
| #2 | C (Concept) | MH "Emotional Intelligence" OR MH "Self-Efficacy" OR MH "Hardiness" OR MH "Social Capital" OR MH "Empathy" OR MH "Empowerment" OR MH "Wit and Humor" OR MH "Coping+" OR MH "Locus of Control" OR TI ( Gratitude OR "Learned resourcefulness" OR "learned optimism" OR "learned hopefulness" OR "Sense of coherence" OR "emotional intelligence" OR "self-efficacy" OR "cultural capital" OR Connectedness OR Hardiness OR "social capital" OR Resilience OR Flourishing OR "Action competence" OR Empathy OR Empowerment OR "Will to meaning" OR Interdisciplinary OR Humour OR Coping OR "locus of control" OR Wellbeing OR "Ecological system theory" OR salutogenesis ) OR AB ( Gratitude OR "Learned resourcefulness" OR "learned optimism" OR "learned hopefulness" OR "Sense of coherence" OR "emotional intelligence" OR "self-efficacy" OR "cultural capital" OR Connectedness OR Hardiness OR "social capital" OR Resilience OR Flourishing OR "Action competence" OR Empathy OR Empowerment OR "Will to meaning" OR Interdisciplinary OR Humour OR Coping OR "locus of control" OR Wellbeing OR "Ecological system theory" OR salutogenesis ) | 223,201 |
| #3 | C (Context) | MH "Periodontal Diseases+" OR MH "Periodontitis+" OR MH "Oral Health" OR MH "Dental Caries" OR TI ("dental caries" OR "tooth decay" OR "oral health" OR "periodontitis" OR "Periodontal Disease") OR AB ("dental caries" OR "tooth decay" OR "oral health" OR "periodontitis" OR "Periodontal Disease") | 54,991 |
| #4 | PCC | #1 AND #2 AND #3 | 438 |

PsycInfo via ProQuest

| **No** | **Description** | **Search string** | **Hits** |
| --- | --- | --- | --- |
| #1 | P (Population) | tiab(adult) | 458,688 |
| #2 | C (Concept) | MAINSUBJECT.EXACT.EXPLODE("Gratitude") OR MAINSUBJECT.EXACT.EXPLODE("Emotional Intelligence") OR MAINSUBJECT.EXACT.EXPLODE("Sense of Coherence") OR MAINSUBJECT.EXACT.EXPLODE("Self-Efficacy") OR MAINSUBJECT.EXACT.EXPLODE("Social Connectedness") OR MAINSUBJECT.EXACT.EXPLODE("Social Capital") OR MAINSUBJECT.EXACT.EXPLODE("Resilience (Psychological)") OR MAINSUBJECT.EXACT.EXPLODE("Empathy") OR MAINSUBJECT.EXACT.EXPLODE("Empowerment") OR MAINSUBJECT.EXACT.EXPLODE("Humor") OR MAINSUBJECT.EXACT.EXPLODE("Coping Behavior") OR MAINSUBJECT.EXACT.EXPLODE("Well Being") OR tiab(Gratitude OR "Learned resourcefulness" OR "learned optimism" OR "learned hopefulness" OR "Sense of coherence" OR "emotional intelligence" OR "self-efficacy" OR "cultural capital" OR Connectedness OR Hardiness OR "social capital" OR Resilience OR Flourishing OR "Action competence" OR Empathy OR Empowerment OR "Will to meaning" OR Interdisciplinary OR Humour OR Coping OR "locus of control" OR Wellbeing OR "Ecological system theory" OR salutogenesis ) | 457,570 |
| #3 | C (Context) | tiab("Dental Caries" OR "tooth decay" OR "oral health" OR periodontitis OR "Periodontal Diseases") OR MAINSUBJECT.EXACT.EXPLODE("Oral Health") | 2,659 |
| #4 | PCC | #1 AND #2 AND #3 | 144 |
