# Supplementary material for: A lens on salutogenic concepts as resources for oral health among adults: a scoping review
Source: BMC Oral Health. 2026 Nov 12;26:2249. doi: 10.1186/s12903-026-10341-2 (PMC13757107; doi:10.1186/s12903-026-10341-2)
Supplement: Supplementary file 2 [file 12903_2026_10341_MOESM2_ESM.docx]

**Studies excluded after full-text screening**

| **Study** | **Title** | **Exclusion reason** |
| --- | --- | --- |
| Adams et al. 2017 | Integrating a Nurse-Midwife-Led Oral Health Intervention Into Centering Pregnancy Prenatal Care: Results of a Pilot Study | Wrong outcomes |
| Agarwal et al. 2021 | Oral health perception and plight of patients of schizophrenia | No age stratification |
| Aida et al. 2011 | Income inequality, social capital and self-rated health and dental status in older Japanese | Wrong age group |
| Amoah et al. 2022 | Association of Health Literacy and Socioeconomic Status with Oral Health Among Older Adults in Ghana: A Moderation Analysis of Social Capital | No age stratification |
| Anagnostopoulos et al. 2011 | Self-efficacy and oral hygiene beliefs about toothbrushing in dental patients: a model-guided study | No age stratification |
| Aurlene et al. 2019 | Does Individual Deprivation Affect the Sense of Coherence and Oral Health Status of Individuals? | No age stratification |
| Bernabé et al. 2012 | Sense of coherence and four-year caries incidence in Finnish adults | No age stratification |
| Bernabé et al. 2012 | Childhood socioeconomic position, adult sense of coherence and tooth retention | No age stratification |
| Bernabé et al. 2010 | Sense of coherence and oral health in dentate adults: findings from the Finnish Health 2000 survey | No age stratification |
| Borges et al. 2014 | Adult tooth loss profile in accordance with social capital and demographic and socioeconomic characteristics | Not english |
| Borkowska et al. 1998 | The relationship of health beliefs and psychological mood to patient adherence to oral hygiene behaviour | Age not specified |
| Borrell et al. 2011 | Social disparities in periodontitis among US adults: the effect of allostatic load | No age stratification |
| Chakraborty et al. 2017 | Estimating peer density effects on oral health for community-based older adults | No age stratification |
| Chaudhary et al. 2021 | The relationship between psychosocial distress and oral health status in patients with facial burns and mediation by oral health behaviour | No age stratification |
| Cinar et al. 2018 | A new complementary approach for oral health and diabetes management: health coaching | No age stratification |
| Cyrino et al. 2016 | Sense of coherence and periodontal health outcomes | No age stratification |
| daSilva & Vettore 2016 | Sense of coherence modifies the association between untreated dental caries and dental pain in low-social status women | No age stratification |
| Davoglio et al. 2016 | Relationship between Sense of Coherence and oral health in adults and elderly Brazilians | No age stratification |
| deOliveiraMiranda et al. 2021 | Sense of coherence and oral health of users of psychoactive substances. | No age stratification |
| deSilva-Sanigorski et al. 2013 | Parental self-efficacy and oral health-related knowledge are associated with parent and child oral health behaviors and self-reported oral health status | Age not specified |
| deSouza et al. 2002 | Self-efficacy as a mediator for improvement in oral health clinical indices | No age stratification |
| doAmaralJúnior et al. 2021 | Social capital and self-reported oral health at baseline of the brazilian longitudinal study of aging | No age stratification |
| Dumitrescu 2007 | Investigating the relationship between self-reported oral health status, oral health-related behaviors, type A behavior pattern, perceived stress and emotional intelligence | Age not specified |
| Dumitrescu et al. 2011 | The relationship between self-reported oral health, self-regulation, proactive coping, procrastination and proactive attitude | Age not specified |
| Dumitrescu & Kawamura 2010 | Involvement of psychosocial factors in the association of obesity with periodontitis | No age stratification |
| Dumitrescu et al. 2009 | Self-reported oral health status, oral health-related behaviours, resilience and hope in Romania | Age range not specified |
| Dumitrescu et al. 2009 | Self-liking, self-competence, body investment and perfectionism: associations with oral health status and oral-health-related behaviours | Age range not specified |
| Dumitrescu et al. 2010 | Relationship of humour with oral health status and behaviours | Age range not specified |
| Finlayson et al. 2011 | Oral health disparities and psychosocial correlates of self-rated oral health in the National Survey of American Life. | No age stratification |
| Folayan et al. 2020 | Psychosocial, education, economic factors, decision-making ability, and caries status of mothers of children younger than 6 years in suburban Nigeria | No age stratification |
| Gao et al. 2014 | Oral health of foreign domestic workers: exploring the social determinants | Wrong outcomes |
| Genco et al. 1999 | Relationship of stress, distress and inadequate coping behaviors to periodontal disease | No age stratification |
| Genco et al. 1998 | Models to evaluate the role of stress in periodontal disease | No age stratification |
| Girestam et al. 2020 | Effects of Domiciliary Professional Oral Care for Care-Dependent Elderly in Nursing Homes - Oral Hygiene, Gingival Bleeding, Root Caries and Nursing Staff's Oral Health Knowledge and Attitudes | Wrong age group |
| Gupta et al. 2015 | Oral Health Inequalities: Relationships between Environmental and Individual Factors | No age stratification |
| Hohlfeld et al. 1989 | Awareness of oral health in 45 to 54-year old Berlin subjects and correlation with clinical periodontal findings] | Not english |
| Holde et al. 2018 | Periodontitis and quality of life: What is the role of socioeconomic status, sense of coherence, dental service use and oral health practices? An exploratory theory-guided analysis on a Norwegian population | No age stratification |
| Ibigbami et al. 2023 | Moderating effects of resilience and self-esteem on associations between self-reported oral health problems, quality of oral health, and mental health among adolescents and adults in Nigeria | No age stratification |
| Islam et al. 2019 | Influence of Occupational Stress and Coping Style on Periodontitis among Japanese Workers: A Cross-Sectional Study | No age stratification |
| James 2020 | The seemingly ‘protective’ effect of internalised racism on overall health among 780 Black/African Americans: The serial mediation of stigma consciousness and locus of control | No age stratification |
| Jamieson et al. 2014 | Self-efficacy and self-rated oral health among pregnant aboriginal Australian women | No age stratification |
| Jones et al. 2016 | Does self-efficacy mediate the effect of oral health literacy on self-rated oral health in an Indigenous population? | No age stratification |
| Jönsson et al. 2020 | The role of psychosocial factors and treatment need in dental service use and oral health among adults in Norway | No age stratification |
| Kanhai et al. 2014 | Sense of coherence and incidence of periodontal disease in adults | No age stratification |
| Khayat et al. 2008 | Perceived stress, coping styles and periodontitis: A cross cultural analysis | No age stratification |
| Kneckt et al. 1999 | Locus of control beliefs predicting oral and diabetes health behavior and health status | No age stratification |
| Kneckt et al. 1999 | Self-efficacy as a common variable in oral health behavior and diabetes adherence | No age stratification |
| Kwate et al. 2014 | An empirical analysis of White privilege, social position and health | No salutogenic concept |
| Laniado et al. 2020 | Social capital and periodontal disease in Hispanic/Latino adults in the United States: Results from the Hispanic Community Health Study/Study of Latinos | No age stratification |
| Lee et al. 2012 | The relationship of oral health literacy and self-efficacy with oral health status and dental neglect | No age stratification |
| Lindmark et al. 2011 | Sense of coherence and oral health status in an adult Swedish population | No age stratification |
| Li et al. 2022 | Association between social capital and oral health among adults aged 50 years and over in China: a cross-sectional study | No age stratification |
| Ludenia et al. 1983 | Dental outpatients: health locus of control correlates | No age stratification |
| Macek et al. 2017 | Oral health conceptual knowledge and its relationships with oral health outcomes: Findings from a Multi-site Health Literacy Study | No age stratification |
| Machado et al. 2017 | Does the Sense of Coherence modifies the relationship of oral clinical conditions and Oral Health-Related Quality of Life? | Wrong age group |
| Merchant et al. 2003 | A prospective study of social support, anger expression and risk of periodontitis in men | No age stratification |
| Meredith et al. 2016 | Associations between adult attachment and: oral health-related quality of life, oral health behaviour, and self-rated oral health | No age stratification |
| Neves et al. 2015 | The Impact of Motivational Interviewing in Reducing Plaque and Bleeding Indices on Probing in Adult Users of the Family Health Strategy | No age stratification |
| Ng & Keung Leung 2006 | A community study on the relationship between stress, coping, affective dispositions and periodontal attachment loss | No age stratification |
| Nishihara et al. 2017 | A periodontal disease care program for patients with type 2 diabetes: A randomized controlled trial | No age stratification |
| Odman et al. 1984 | Utilization of locus of control in the prediction of patients' oral hygiene performance | No age stratification |
| Olutola et al. 2012 | Socio-environmental factors associated with self-rated oral health in South Africa: a multilevel effects model | No age stratification |
| Omori et al. 2019 | Quasi-Randomized Trial of Effects of Perioperative Oral Hygiene Instruction on Inpatients with Heart Diseases Using a Behavioral Six-Step Method | Wrong age group |
| Parker et al. 2022 | Self-efficacy and oral health outcomes in a regional Australian Aboriginal population | No age stratification |
| Parker et al. 2022 | Oral health-related self-efficacy and fatalism in a regional South Australian Aboriginal population | No age stratification |
| Peker & Bermek 2011 | Oral health: locus of control, health behavior, self-rated oral health and socio-demographic factors in Istanbul adults | No age stratification |
| Quinonez 2016 | Psychosocial pathway is key in explaining the relationship between socioeconomic status and oral health | Not original study |
| Radafshar et al. 2012 | Relationships of Stress and Coping Styles to Periodontal Disease: A Case-Control Study. | No age stratification |
| Rawlinson et al. 2021 | Periodontal treatment, psychological factors and oral health‐related quality of life. | No age stratification |
| Rouxel et al. 2015 | Is Social Capital a Determinant of Oral Health among Older Adults? Findings from the English Longitudinal Study of Ageing | No age stratification |
| Rouxel et al. 2015 | Social Capital and Oral Health Among Adults 50 Years and Older: Results From the English Longitudinal Study of Ageing | No age stratification |
| Sánchez et al. 2009 | Dental beliefs in HIV+ patients with different oral health care needs | Age range not specified |
| Sanders et al. 2008 | Resilience to urban poverty: theoretical and empirical considerations for population health | No age stratification |
| Sanders & Spencer 2005 | Why do poor adults rate their oral health poorly? | No age stratification |
| Santiago et al. 2013 | Social capital and dental pain in Brazilian northeast: a multilevel cross-sectional study | No age stratification |
| Santiago et al. 2014 | The relationship between neighborhood empowerment and dental caries experience: a multilevel study in adolescents and adults | No age stratification |
| Savolainen et al. 2009 | Sense of coherence associates with oral and general health behaviours | No age stratification |
| Serrano et al. 2010 | Relationship among metabolic control, oral parameters and self-efficacy in diabetic patients. | No age stratification |
| Soares et al. 2020 | The utility of network analysis in the context of Indigenous Australian oral health literacy | Wrong outcomes |
| Sun et al. 2023 | Psychological factors and oral health during initial outbreak of COVID-19 in China: A cross-sectional study | Age not specified |
| Syrjälä et al. 2004 | Relation of different measures of psychological characteristics to oral health habits, diabetes adherence and related clinical variables among diabetic patients | No age stratification |
| Teixeira et al. 2015 | Association between resilience and quality of life related to oral health in the elderly | Wrong age group |
| Vatne et al. 2015 | Patients' perception of own efforts versus clinically observed outcomes of non-surgical periodontal therapy in a Norwegian population: an observational study | No age stratification |
| Viterbo et al. 2019 | Implementation of an Interdisciplinary Approach to Promote Workers Global Health Status in the Oil Industry, Brazil (2006-2015) | No age stratification |
| Watson et al. 2008 | The influence of active coping and perceived stress on health disparities in a multi-ethnic low income sample | No age stratification |
| Wimmer et al. 2005 | Coping with stress: its influence on periodontal therapy | No age stratification |
| Wu et al. 2020 | Immigrant Status, Resilience, and Perceived Oral Health Among Chinese Americans in Hawaii | No age stratification |
| Ylostalo et al. 2003 | Optimism and life satisfaction as determinants for dental and general health behavior - Oral health habits linked to cardiovascular risk factors | Wrong outcomes |
| Zakershahrak et al. 2023 | Effect of personality traits on socioeconomic inequalities in health, a population-based study | No age stratification |
| Zini et al. 2016 | Validation of an innovative instrument of Positive Oral Health and Well-Being (POHW) | No age stratification |
